# Shaping neuroplasticity by using powered exoskeletons in patients with stroke: a randomized clinical trial

**DOI:** 10.1186/s12984-018-0377-8

**Published:** 2018-04-25

**Authors:** Rocco Salvatore Calabrò, Antonino Naro, Margherita Russo, Placido Bramanti, Luigi Carioti, Tina Balletta, Antonio Buda, Alfredo Manuli, Serena Filoni, Alessia Bramanti

**Affiliations:** 1 IRCCS Centro Neurolesi “Bonino-Pulejo”, S.S. 113, Contrada Casazza, 98124 Messina, Italy; 2 Fondazione Centri di Riabilitazione, P. Pio – Onlus, Lecce, Italy

**Keywords:** Ekso™, Wearable exoskeleton, Plasticity, Stroke recovery, Effective connectivity

## Abstract

**Background:**

The use of neurorobotic devices may improve gait recovery by entraining specific brain plasticity mechanisms, which may be a key issue for successful rehabilitation using such approach. We assessed whether the wearable exoskeleton, Ekso™, could get higher gait performance than conventional overground gait training (OGT) in patients with hemiparesis due to stroke in a chronic phase, and foster the recovery of specific brain plasticity mechanisms.

**Methods:**

We enrolled forty patients in a prospective, pre-post, randomized clinical study. Twenty patients underwent Ekso™ gait training (EGT) (45-min/session, five times/week), in addition to overground gait therapy, whilst 20 patients practiced an OGT of the same duration. All individuals were evaluated about gait performance (10 m walking test), gait cycle, muscle activation pattern (by recording surface electromyography from lower limb muscles), frontoparietal effective connectivity (FPEC) by using EEG, cortico-spinal excitability (CSE), and sensory-motor integration (SMI) from both primary motor areas by using Transcranial Magnetic Stimulation paradigm before and after the gait training.

**Results:**

A significant effect size was found in the EGT-induced improvement in the 10 m walking test (d = 0.9, *p* < 0.001), CSE in the affected side (d = 0.7, *p* = 0.001), SMI in the affected side (d = 0.5, *p* = 0.03), overall *gait quality* (d = 0.8, *p* = 0.001), hip and knee muscle activation (d = 0.8, *p* = 0.001), and FPEC (d = 0.8, *p* = 0.001). The strengthening of FPEC (*r* = 0.601, *p* < 0.001), the increase of SMI in the affected side (*r* = 0.554, *p* < 0.001), and the decrease of SMI in the unaffected side (*r* = − 0.540, *p* < 0.001) were the most important factors correlated with the clinical improvement.

**Conclusions:**

Ekso™ gait training seems promising in gait rehabilitation for post-stroke patients, besides OGT. Our study proposes a putative neurophysiological basis supporting Ekso™ after-effects. This knowledge may be useful to plan highly patient-tailored gait rehabilitation protocols.

**Trial registration:**

ClinicalTrials.gov, NCT03162263.

## Background

Most of the patients with stroke experience a restriction of their mobility. Gait impairment after stroke mainly depends on deficits in functional ambulation capacity, balance, walking velocity, cadence, stride length, and muscle activation pattern, resulting in a longer gait cycle duration and lower than normal stance/swing ratio in the affected side, paralleled by a shorter gait cycle duration and a higher than normal stance/swing ratio in the unaffected side [1].

Conventional gait training often offers non-completely satisfactory results. Specifically, patients with stroke receiving intensive gait training with or without body weight support (BWS) may not improve in walking ability more than those who are not receiving the same treatment (with the exception of walking speed and endurance) [2–5]. Moreover, only patients with stroke who are able to walk benefit most from such an intervention [2–5]. Therefore, there is growing effort to increase the efficacy of gait rehabilitation for stroke patients by using advanced technical devices. Neurorobotic devices, including robotic-assisted gait training (RAGT) with BWS, result in a more likely achievement of independent walking when coupled with overground gait training (OGT) in patients with stroke. Specifically, RAGT combined with OGT has an additional beneficial effect on functional ambulation outcomes, although depending on the duration and intensity of RAGT [6, 7]. Further, RAGT requires a more active subject participation in gait training as compared to the traditional OGT, which is a vital feature of gait rehabilitation [7, 8].

Even though no substantial differences have been reported among the different types of RAGT devices [9], a main problem with neurorobotic devices is the provision for the patient of a real-world setting ambulation [10, 11]. To this end, wearable powered exoskeletons, e.g., the Ekso™ (Ekso™ Bionics, Richmond, CA, USA), have been designed to improve OGT in neurologic patients.

Notwithstanding, the efficacy of wearable powered exoskeletons in improving functional ambulation capacity (including gait pattern, step length, walking speed and endurance, balance and coordination) has not been definitively proven, and any further benefit in terms of gait performance remains to be confirmed. However, a recent study showed that Ekso™ could improve functional ambulation capacity in patients with sub-acute and chronic stroke [12]. Therefore, a first aim of our study was to assess whether Ekso™ is useful in improving functional ambulation capacity and gait performance in chronic post-stroke patients compared to conventional OGT.

The neurophysiologic mechanisms harnessed by powered exoskeletons to favor the recovery of functional ambulation capacity are still unclear. It is argued that the efficacy of neurorobotics in improving functional ambulation capacity depends on the high frequency and intensity of repetition of task-oriented movements [13]. This could guarantee a potentially stronger entrainment of the neuroplasticity mechanisms related to motor learning and function recovery following brain injury, including sensorimotor plasticity, frontoparietal effective connectivity (FPEC), and transcallosal inhibition, as compared to conventional therapy [14–16]. Moreover, the generation and strengthening of new connections supporting the learned behaviors, and the steady recruitment of these neural connections as preferential to the learned behaviors occur through these mechanisms, thus making the re-learned abilities long lasting [13, 14, 17–23].

Such neurophysiologic mechanisms have been tested in neurorobotic rehabilitation using stationary exoskeletons (e.g. Lokomat™) [13, 14]. Therefore, the second aim of our study was to assess whether there are specific neurophysiological mechanisms (among those related to sensorimotor plasticity, FPEC, and transcallosal inhibition) by which Ekso™ improves functional ambulation capacity in the chronic post-stroke phase. The importance of knowing these mechanisms is remarkable in order to implement patient-tailored rehabilitative training, given that any further advance in motor function recovery mainly relies on motor rehabilitation training, whereas spontaneous motor recovery occurs within 6 months of a stroke [24]*.* This is also the reason why we focused our study on patients with chronic stroke.

## Methods

### Patients

Eligible patients were selected among those who attended our Neurorobotic Rehabilitation Unit between May and August, 2017. They had to be aged ≥55 years (so as to avoid cases of young stroke), suffering from a first, single ischemic supra-tentorial stroke (that is a simple and basic model to study plasticity mechanisms following a stroke) occurred more than six months before the study inclusion, with a Muscle Research Council score of ≤3, a Mini-Mental State Examination of > 24, a Modified Ashworth Scale, MAS, of ≤2 of muscles of hip, knee, and ankle, and a Functional Ambulatory Categories of ≤ 4. Moreover, they had to meet the inclusion/exclusion criteria of the manufacturer’s recommendations. Clinic-demographic characteristics are reported in Table 1. The study was approved by our local Ethics Committee and was registered in ClinicalTrials.gov, NCT03162263. All participants gave their written informed consent to individual patient data reporting before participating in the study.

**Table 1:** Shows the individual clinical-demographic characteristics

group	Age (y)	gender	lesion location	stroke onset (m)	comorbidities
EGT (*n* = 20)	67	M	r FP	8	3
	68	F	l PO	11	3
	70	M	r TP	9	1
	59	F	l PO	11	1 + 3
	64	M	r FP	6	none
	72	F	r P	10	1 + 2
	74	M	l F	7	2
	69	M	r FP	6	3
	66	F	l PO	11	2
	70	M	r FP	14	4
	73	M	r FP	10	1
	69	F	l PO	10	4
	67	M	r TP	6	2
	74	F	l PO	8	2
	67	M	r FP	14	none
	67	F	r P	12	2
	70	M	l F	13	1 + 2
	71	M	r FP	10	4
	71	F	l PO	8	2
	68	M	r FP	8	4
mean ± SD	69 ± 4	12 M,8F		10 ± 3	
OGT (*n* = 20)	65	M	r P	12	1
	61	F	l F	10	1 + 4
	66	M	l F	8	3 + 4
	77	F	r P	11	2
	55	M	r FP	10	4
	68	F	r TP	6	5
	66	M	l TP	11	none
	69	M	r P	14	3
	73	F	l F	12	2
	66	M	r P	9	none
	66	F	l F	13	3
	57	M	l F	12	2
	71	F	r P	9	5
	70	M	r FP	9	2
	60	F	r TP	14	3
	72	M	l TP	6	none
	64	M	r P	12	3
	75	F	l F	6	1 + 3
	63	M	r P	14	5
	66	F	l F	12	none
mean ± SD	67 ± 6	11 M,9F		11 ± 3	
Z	0.2	0.7	0.6	0.3	0.5

*Legend*: *EGT Ekso*™ gait training, *OGT* overground gait training, *F* frontal, *P* parietal, *O* occipital, *T* temporal, *l* left, *r* right, *1* high blood pressure, *2* diabetes mellitus, *3* hypercholesterolemia, *4* smoking, *5* alcoholism. Z Z-Score calculator for 2 population proportions

### Study design

**Fig. 1:**
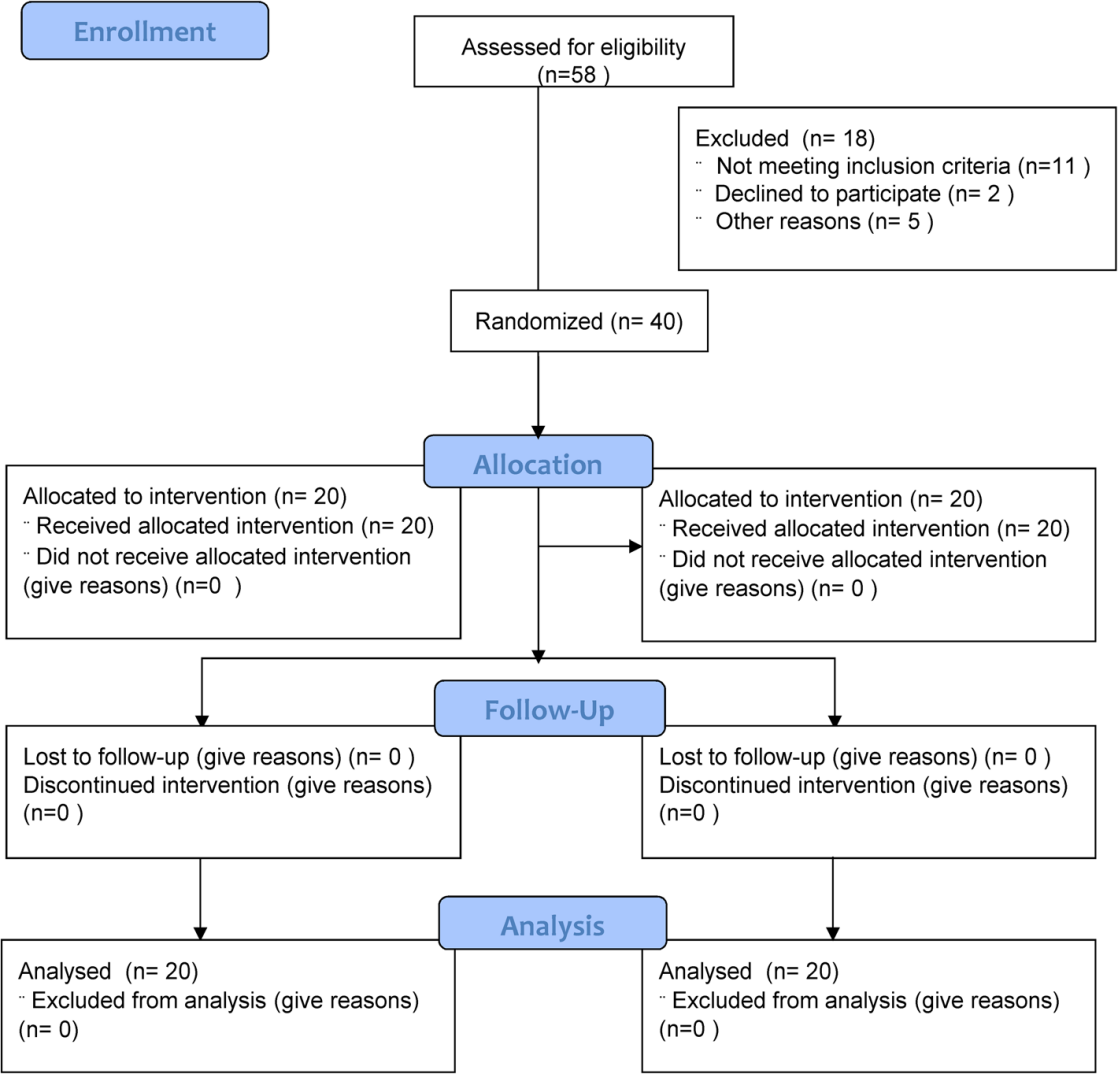
Experimental study flow diagram

The present study was designed as a randomized clinical trial (prospective, assessor blinded, parallel group study). Forty out of 58 outpatients attending the Neurorobotic Rehabilitation Unit of our Institute were rated as eligible according to the abovementioned inclusion and exclusion criteria and were included in the study (Fig. 1).

The sample size estimate was based on extrapolations from previous studies examining the effects of exoskeletons on gait in patients with stroke [25–29]. Accordingly, we used the effect size (0.9) of the primary composite endpoint for calculations. Power was set at 80%, alpha at 5%; we accounted for a dropout rate of 10%. Using a relatively conservative estimation, a total of 40 subjects (20 per arm) would be required to detect a difference in the primary outcome getting the Minimally Clinically Important Difference (MCID) at the end of the training, assuming non-inferiority with moderate correlations among covariates (R-squared = 0.5).

The enrolled patients were equally randomized into the EGT or the OGT group, with a 1:1 allocation ratio. For randomization, sealed envelopes were prepared in advance and marked on the inside with a + (EGT) or – (OGT). Both the groups were provided with conventional physiotherapy training (including a 15-min warm-up and cool-down period), scheduled in five sessions per week for eight consecutive weeks, 60 min for each session. In addition to conventional physiotherapy training, EGT patients practiced 45-min session of Ekso™ training, while OGT patients underwent 45-min of conventional gait training, for all 8 weeks.

Before the training (TPRE), we evaluated some clinical parameters (10-m walk test, 10MWT, Rivermead Mobility Index, RMI, and timed up and go test, TUG), gait pattern (by recording surface electromyography –sEMG- from lower limbs), FPEC by using EEG, corticospinal excitability (CSE) and sensory-motor integration (SMI) by using Transcranial Magnetic Stimulation (TMS) paradigm over the affected and unaffected hemisphere. All these measures were repeated after the end of the gait training (TPOST), i.e., 8 weeks after starting the training. The experimenters who collected the various measures of plasticity, those who analyzed the data, and the therapists who performed the clinical tests were blind to patient allocation.

### Gait training

**Fig. 2:**
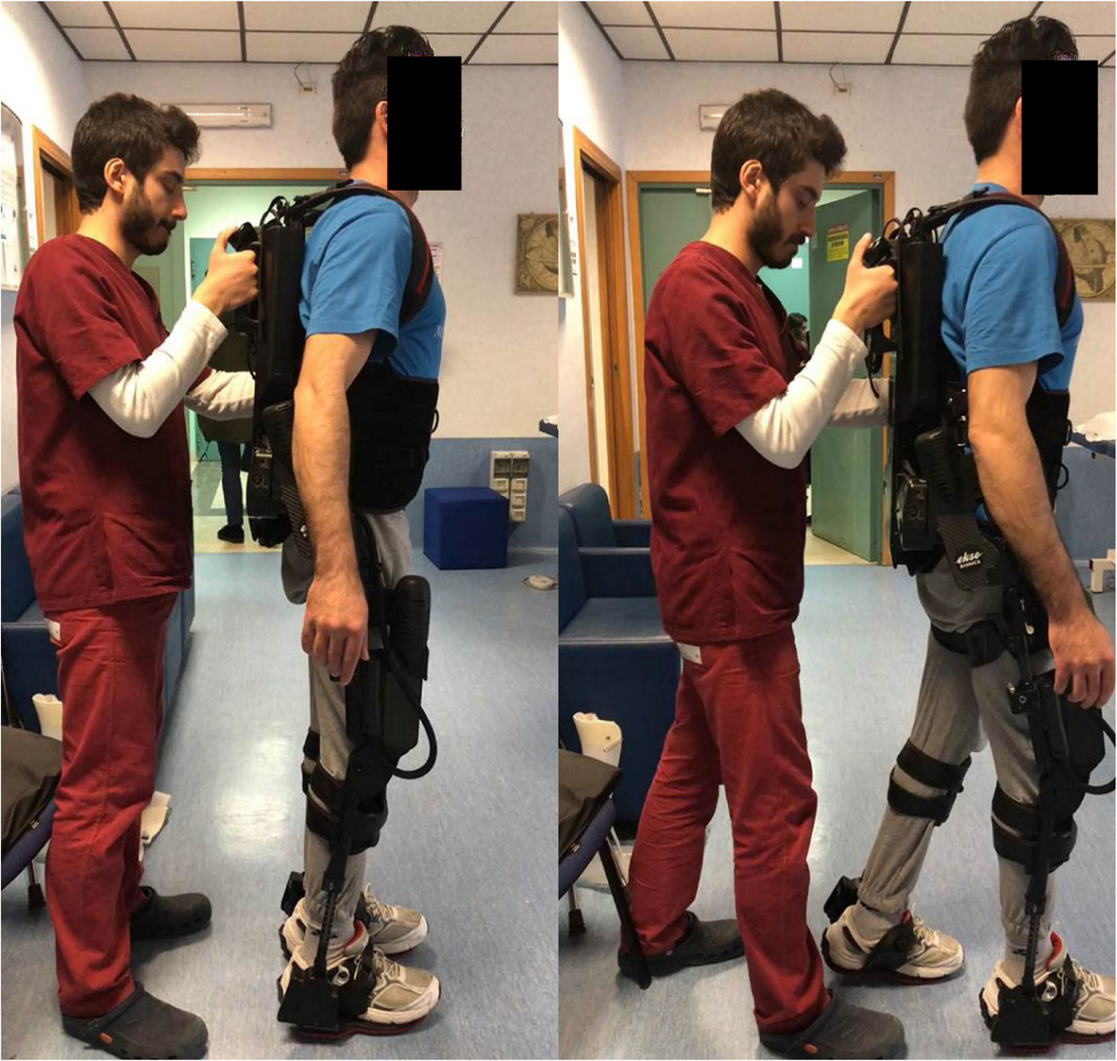
Ekso™ device

Ekso™ is an exoskeleton framework for the lower limbs, equipped with (1) electric motors to power movement for the hip and knee joints, (2) passive spring-loaded ankle joints, (3) foot plates on which the user stands, and (4) a backpack that houses a computer, battery supply, and wired controller (Fig. 2). A rigid backpack is an integral structural component of the exoskeleton, which provides support from the posterior pelvis to the upper back, besides carrying the computer and batteries. The exoskeleton attaches to the user’s body with straps over the dorsum of the foot, anterior shin and thigh, abdomen, and anterior shoulders. The limb and pelvic segments are adjustable to the user’s leg and thigh length, and the segment across the pelvis is adjustable for hip width and hip abduction angle.

The user can stand up, sit down, and walk with the help of a front-wheeled walker and with the exoskeleton attached to a ceiling rail tether. A physical therapist initially provides assistance to maintain the user’s center of mass over the base of support to prevent falling (Fig. 2). At first, steps are initiated one at a time by the instructor, as the user is guided to a position of stance on one foot. The onboard computer coordinates the knee and hip movement needed, given the user’s physical size characteristics, to achieve the desired step. As the user learns to weight-shift to a stance position, the exoskeleton can be set to trigger steps automatically when the user hits preset targets for forwarding and lateral weight shifts onto the stance leg. Users also progress from standing up, walking, and sitting down with a front-wheeled walker to using Lofstrand crutches. Over time, instructors reduce the level of assistance they provide and increase the duration of walking during a session. An Ekso™-trained physiotherapist supervised patients’ cooperation and participation in the treatment.

The OGT group underwent sessions of assisted over-ground walking. The physiotherapist oversaw the entire session, guiding the patient to travel along the same walking lane used for the EGT group, using their habitual walking device (crutches, rollator), and shoes, and maintaining the same velocity with the Ekso™ device.

### Outcome measures

The primary goal was to obtain an improvement in lower limb gait and balance at the end of the training getting the MCID for the 10MWT, RMI, and TUG scales (composite primary outcome). Secondary outcomes consisted of the modifications of FPEC, CSE, and SMI magnitude, overall gait quality, and hip and knee muscle activation.

### TMS paradigm

CSE and SMI were probed using TMS pulses with a monophasic pulse configuration and peripheral nerve electric stimuli. Magnetic pulses were delivered to the affected and unaffected leg-M1 [30] using a standard figure-of-eight coil (diameter of each wing, 90 mm) connected with a high-power Magstim200 stimulator (Magstim Co, Ltd.; UK). The intensity of TMS pulses was adjusted to evoke a muscle response in relaxed abductor hallucis (AH) muscle with a peak-to-peak amplitude of approximately 0.5 mV [31]. SMI was studied using the conditioning-test protocol described by Bikmullina and colleagues [32]. Conditioning electrical stimuli (1 ms in duration and a stimulation intensity of 2.5 times the individual’s sensory perception threshold) were delivered to great toe and preceded the TMS test pulses to contralateral leg-M1 of 55 ms. The mean amplitude of 10 conditioned-MEP was expressed as a percentage of the mean amplitude of 10 unconditioned MEP. Then, we applied a 1 Hz-rTMS protocol over the unaffected M1-leg (1000 pulses at an intensity of 90% of the RMT from AH) [33], using the above-mentioned TMS setup. The overall modulation of MEP and SMI amplitude (from both hemispheres) induced immediately (T0), 30 min (T30), and 60 min (T60) after the rTMS protocol application was calculated as the ratio between the maximum and mean value (among T0, T30, and T60) and taken as a measure of CSE modulation.

### Effective connectivity

EEG was recorded using a high-input impedance amplifier (referential input noise < 0.5μVrms @ 1÷20,000 Hz, referential input signal range 150-1000mVPP, input impedance >1GΩ, CMRR > 100 dB, 22bit ADC) of Brain Quick SystemPLUS (Micromed; Mogliano Veneto, Italy), wired to an EEG cap equipped with 21 Ag tin disk electrodes, positioned according to the international 10-20 system. An electrooculogram (EOG) (0.3-70 Hz band-pass) was also recorded. The recording occurred in the morning (about 11^am^) and lasted at least 10 min, with the eyes open (fixing a point in front of the patient). The EEG end EOG were sampled at 512 Hz, filtered at 0.3-70 Hz, and referenced to linked earlobes [34].

EEG recordings contaminated by blinking, eye movements, movements, and other artifacts were rejected off-line by visual inspection and based on independent component analysis (ICA) data.

First, we identified the cortical activations induced by gait training from the EEG recordings by using Low-Resolution Brain Electromagnetic Tomography (LORETA; free release of LORETA-KEY alpha-software) [35–39].

The brain compartment of the three-shell spherical head model used in the LORETA was restricted to the cortical gray matter, Talairach co-registered [28], and had a resolution of 7 mm, thus obtaining 2394 voxels (i.e., equivalent current dipoles). Therefore, it can be assumed that localization accuracy is at worst in the order of 14 mm, given the 7 mm resolution of the current implementation of LORETA-Talairach with 21 electrodes [40]. The voxels of LORETA solutions were collapsed in 7 regions of interest (ROIs) (prefrontal, PF, supplementary motor, SMA, centroparietal, CP, and occipital, O, areas of both hemispheres) [41] determined according to the brain model coded into Talairach space, by using MATLAB.

Then, structural equation modeling (SEM) technique (or path analysis) was employed to measure the effective connectivity (that assesses the causal influence that one brain area, i.e., electrode-group, exerts over another, under the assumption of a given mechanistic model) [42, 43] among the cortical activations induced by gait trainings. SEM combines a network model supporting the putative connections linking sets of cortical activations and the inter-regional covariances of activity (i.e., the degree to which the activities of two or more regions, i.e., electrode-group, are related), to estimate the influence of one region (electrode set) on another through the putative connections linking the sets of (electrode) activation [41]. The network model, supporting inter-regional connectivity employed in our study, was defined according to a previous study and included the abovementioned ROIs [41]. The SEM model that was used began with zero paths and, from-time-to-time, added paths to improve the model’s stability until no further improvement in the model’s stability was found [44]. Therefore, the SEM generates linear equations that describe the relationship between the variables of interest (i.e., ROI activations), which can be described as paths (i.e., vectors indicating the direction of influence) and path-coefficients (i.e., values reflecting the strength of influence). In other words, an *x → y* path-coefficient indicates by how many SD units the *y* increases, corresponding to an increase of 1 SD unit of the *x* [45–48].

### Gait data analysis

An eight-channel wireless sEMG device (BTS; Milan, Italy) was used to record the EMG activity (sampled at 1 kHz, filtered at 5-300 Hz) from eight muscles (both tibialis anterior -TA, soleus -S, rectus femoris -RF, and biceps femoris -BF). The device was also equipped with an accelerometer, which was set at lumbar level, to establish the gait phases. Gait analysis was conducted on a 10-m walkway and two gaits at a self-selected speed were collected. The following gait parameters for both the affected and unaffected lower limbs were measured [49]: (i) step cadence (number of steps per minute); (ii) gait cycle duration (time from one right heel strike -initial contact- to the next one -end of terminal swing); (iii) stance/swing ratio (ratio between stance from heel strike to toe-off, and swing phase duration from toe-off to heel strike); and (iv) the gait quality index, i.e., an overall gait performance score, reflecting an approximate 60:40% distribution of stance:swing phases. The unaffected and affected side values for each parameter were averaged from the two 10-m gaits and were used for subsequent analyses. In addition, the standard deviations (SD) of the gait variables for each participant were also obtained as a measure of gait variability. All parameters were measured before and after the gait training. The EMG signal was analyzed for root-mean-square (RMS) (a temporal parameter estimating muscle activation) to investigate lower-limb muscle activation modified by gait training [50].

### Data analysis

Normal distribution and homogeneity of variance of data were assessed by using the Shapiro–Wilks and Levene test, respectively. Baseline differences were assessed by using *t-*tests. Gait training induced changes in any outcome measure were explored by repeated measures ANOVA or by the Wilcoxon test (W), where appropriate, with the factors *group* (two levels: EGT and OGT), *time* (two -TPRE and TPOST- or four levels –TPRE/TPOST, T0, T30, and T60), *path*-*direction* and *path-coefficients* (49 levels). A *p*-value < 0.05 was considered significant. Conditional on a significant *F-*value, *post-hoc t-*tests were performed with Bonferroni correction for multiple comparisons (α = 0.0055). Descriptive analysis was used to evaluate the effect size measures between the two independent groups (Cohen’s *d* calculation, p-value). Last, we implemented a multiple logistic regression model to calculate the prognostic accuracy of each electrophysiological outcome measure in the prediction of clinical recovery, considering composite primary outcome measure improvement as a dependent variable and electrophysiological outcome measures as predictive (independent) variables.

## Results

All participants completed the training without any significant adverse events, except a mild skin bleachable erythema at the thigh and shank strap locations in seven patients of the EGT. Baseline data were normally distributed (*p* > 0.2) and homogeneous in variance (*p* > 0.1). Moreover, the two groups showed non-significant baseline differences concerning clinical-demographic, biomechanical, and electrophysiological parameters (all comparisons *p* > 0.05).

**Fig. 3:**
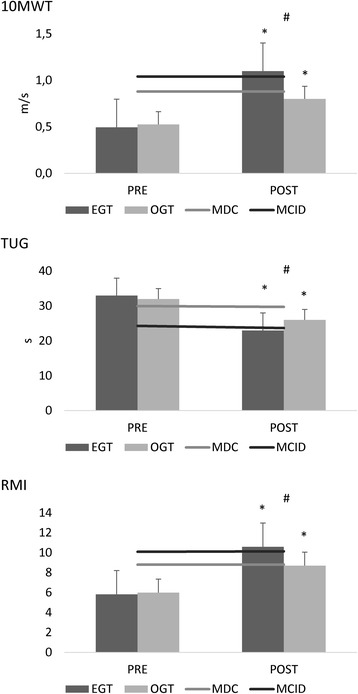
Primary outcome measures (10MWT 10 m walk test, RMI Rivermead Mobility Index, TUG timed up and go test) assessed at TPRE and TPOST in the two groups (EGT and OGT). Minimally Clinically Important Difference (MCID) and Minimal Detectable Change (MDC) are reported as well. * refer to *post-hoc p*-values of within-group analysis (significant whether *p* < 0.016), whereas # refer to p-values of between-group analysis for TPOST-TPRE difference (*p* < 0.05). Vertical error bars refer to SD

**Fig. 4:**
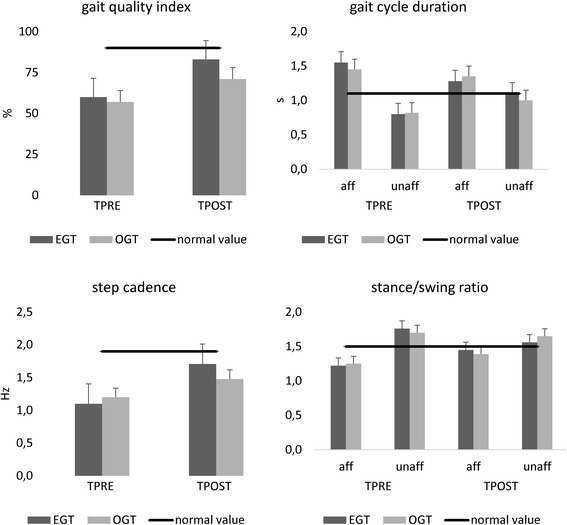
Mean gait parameters of the affected and unaffected lower limbs at baseline (TPRE) and after gait training (TPOST) in Ekso™ (EGT) and overground gait training (OGT). Normative values are reported as well (black horizontal lines). * refer to p-values of within-group analysis (significant whether *p* < 0.008), whereas # refer to p-values of between-group analysis for TPOST-TPRE difference (p < 0.05). Vertical error bars refer to SD

**Fig. 5:**
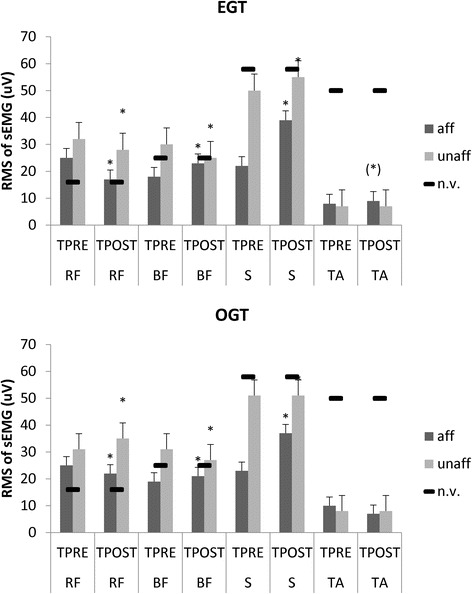
Mean muscle activity of the paretic (aff) and non-paretic (unaff) muscles (TA tibialis anterior; S soleus; RF rectus femoris; BF biceps femoris) during gait at baseline (TPRE) and after gait training (TPOST) in EGT and OGT. Normative values are reported as well (black horizontal lines). Vertical error bars refer to SD

Both groups showed a reduced gait velocity, a high TUG, and a low RMI score, compared to the normative values from healthy controls [51, 52] (Fig. 3). In parallel, they got a low overall gait quality index, a longer than normal gait cycle duration and lower than normal stance/swing ratio in the affected side, paralleled by a shorter than normal gait cycle duration and a higher than normal stance/swing ratio in the unaffected side (Fig. 4). The alteration in velocity, stride length, and cadence were related to the abnormal average RMS values. We found a higher than normal activation of RF (more in the unaffected than affected side), a lower than normal activation of affected BF, a higher than normal activation of unaffected BF, a much lower than normal activation of S (more in the affected than unaffected side), and a lower than normal activation of both TA (Fig. 5). Altogether, these abnormalities in muscle activation led to reduced hip, knee, and ankle flexion during the swing phase, and a decreased extension of the hip during the stance phase.

**Fig. 6:**
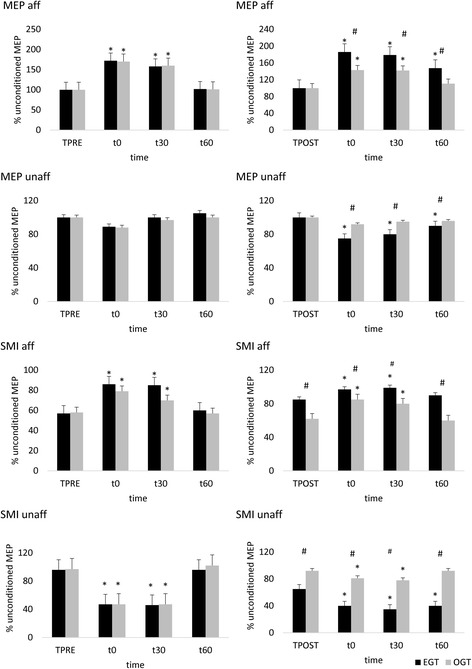
rTMS outcome measures assessed at TPRE and TPOST in the two groups (EGT and OGT). Left and right columns illustrate the rTMS findings (MEP -motor evoked potential- and SMI -sensory-motor integration- in the affected -aff- and unaffected -unaff- hemispheres) before and after gait training, respectively. * refer to *post-hoc p*-values of within-group analysis (significant whether p < 0.008), whereas # refer to p-values of between-group analysis for TPOST-TPRE difference (p < 0.05). Vertical error bars refer to SD

Regarding neurophysiologic measures at baseline, we found a low MEP amplitude paralleled by a high SMI strength (i.e., conditioned MEP amplitude decrease) in the affected hemisphere, and a low SMI strength (i.e., conditioned MEP amplitude increase) in the unaffected hemisphere (Fig. 6). The rTMS had weak aftereffects, i.e., a mild MEP amplitude increase and a SMI strength decrease in the affected hemisphere, and a mild MEP amplitude decrease and a SMI strength increase in the unaffected hemisphere (Fig. 6). Finally, brain connectivity was characterized by a globally deteriorated effective connectivity among PF, SMA, and CP, paralleled by a hyperconnectivity among CP, O, and SMA (Fig. 8).

All EGT patients met the composite primary outcome, i.e., getting the MCID for the 10MWT, RMI, and TUG scales at the end of the training (reflecting an improvement in lower limb gait and balance). This was not the case of OGT. Further, patients belonging to EGT got the Minimal Detectable Change (MDC) in each outcome measure, whereas those belonging to OGT did not get the MDC for 10MWT (Fig. 3) (*time×group* interaction 10MWT F_(1,38)_ = 13, *p* = 0.001; TUG F_(1,38)_ = 3.5,*p* = 0.04; RMI F_(1,38)_ = 4.4, p = 0.04). Specifically, there was a large effect size for 10MWT (d = 0.9, *p* < 0.001) and a mild-to-moderate one for TUG (0.5, *p* = 0.02) and RMI (0.6, *p* = 0.03). Indeed, the EGT showed greater changes than OGT, and got the MCID for every primary outcome measure, differently from OGT (Fig. 3).

The more evident clinical improvement in EGT patients was paralleled by a similar enhancement of the gait parameters. Specifically, the gait quality index (*time×group* F_(1,38)_ = 43, *p* < 0.001, d = 0.9) and the step cadence (*time×group* F_(1,38)_ = 17, *p* < 0.001, d = 0.9) improved more in the EGT than OGT (Fig. 4). Also, we observed a more evident reduction of the gait cycle duration (*time×group* F_(1,38)_ = 17, *p* < 0.001, d = 0.9) and a more evident increase in stance/swing ratio (*time×group* F_(1,38)_ = 8.6, *p* = 0.008, d = 0.8) in the affected limb in EGT than OGT. In parallel, we found a more evident shortening of gait cycle duration (*time×group* F_(1,38)_ = 12, *p* < 0.001, d = 0.9) and a decrease of stance/swing ratio in the unaffected limb (*time×group* F_(1,38)_ = 14, *p* < 0.001, d = 0.9) in EGT than OGT.

These changes were associated to the modification of muscle activation (Fig. 5). Gait training significantly modified the EMG averaged amplitudes of both limbs (*time×muscle×side×group* F_(3,114)_ = 6.8, p < 0.001) but with specific differences among groups and muscles. In particular, the EMG amplitude of paretic muscles over the entire gait cycle at TPOST were affected more in EGT (*time×muscle* F_(3,57)_ = 4.3, *p* = 0.007, d = 0.8) than OGT group (*time×muscle* F_(3,57)_ = 2.8, *p* = 0.04, d = 0.6), as compared to the non-paretic ones. Specifically, we found a significant RMS decrease in the affected and unaffected RF and the unaffected BF, and a magnitude increase in the affected BF and the affected and unaffected S (Fig. 5; *post-hoc p-*values significant when *p* < 0.008). Both TA muscles showed non-significant changes, except a trend to an increased activation in the paretic TA muscle in the EGT group (*p* = 0.01).

**Fig. 7:**
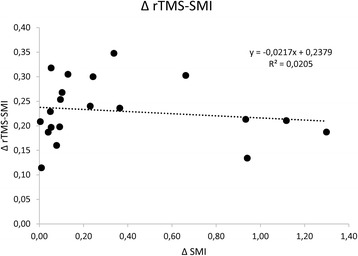
Shows that the difference in sensory-motor integration (SMI) between the two groups at baseline was not correlated with rTMS-induced SMI aftereffects

Last, we probed sensorimotor plasticity and CSE by means of TMS to assess whether EGT may harness one or more of the abovementioned mechanisms of plasticity to induce clinical-biomechanical changes. Following rTMS, MEP amplitude in the affected hemisphere increased more in EGT than OGT (*time×group* F_(3,114)_ = 5.7, *p* = 0.001; d = 0.8), whilst SMI strength equally decreased (i.e., conditioned MEP amplitude increased) in both groups (*time×group p* = 0.4), given that SMI strength was different between the groups already at the TPOST baseline. MEP amplitude in the unaffected hemisphere slightly decreased only in the EGT group (*time×group* F_(3,114)_ = 4, *p* = 0.01, d = 0.6), whilst SMI strength increased more (i.e., conditioned MEP amplitude decreased) in EGT than OGT (*time×group* F_(3,114)_ = 5.8, *p* = 0.001, d = 0.8), despite SMI strength being different between the groups already at the TPOST baseline (Fig. 6). Anyway, such a baseline difference was not correlated with the magnitude of rTMS aftereffects (Fig. 7). Altogether, EGT induced a rebalance of the SMI of the affected and unaffected hemispheres, in parallel to CSE interhemispheric remodulation, whereas OGT acted more on the affected than unaffected hemisphere. The *post-hoc t-*tests are summarized in Fig. 5 (significant whether *p* < 0.008).

**Fig. 8:**
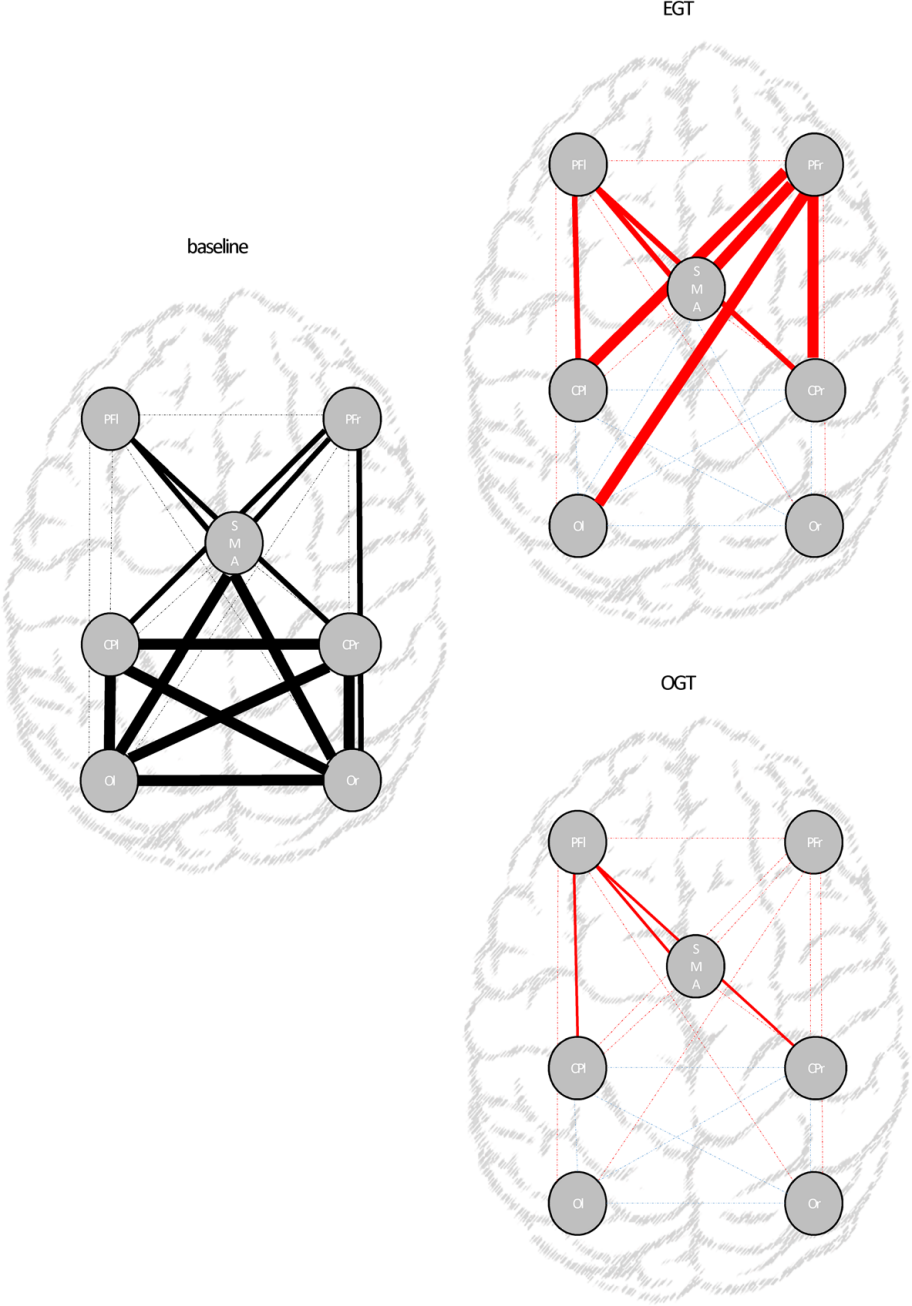
Illustrates the connectivity paths at baseline and following gait training (EGT and OGT). Red color indicates a path-coefficient increase (significant whether *p* < 0.0001), while blue color a decrease at TPOST as compared to TPRE. Line thickness indicates whether the TPOST-TPRE changes were detectable only following EGT –thick-, greater following EGT than OGT –medium- or equally significant in both groups –thin. Legend: l left hemisphere; O occipital areas; CP centroparietal areas; PF prefrontal areas; r right hemisphere; SMA supplementary motor area

Effective connectivity data are summarized in Fig. 8. A three-way ANOVA analysis returned a significant *time×group×path* interaction (F_(48,1824)_ = 1.8, p = 0.001, d = 0.6), suggesting that both groups modified FPEC but differently in extent and time. In detail, only EGT increased PF-SMA connectivity in the unaffected hemisphere (*time×group* F_(1,38)_ = 4.6, *p* = 0.03, d = 0.7), the ipsilateral (*time×group* F_(1,38)_ = 18, *p* < 0.001, d = 0.9) and contralateral PF-P (*time×group* F_(1,38)_ = 8.5, *p* = 0.006, d = 0.8) and the contralateral PF-O connectivity within the unaffected hemisphere (*time×group* F_(1,38)_ = 7.8, *p* = 0.008, d = 0.9). EGT induced a more evident improvement in ipsilateral and contralateral PF-CP (*time×group* F_(1,38)_ = 13, p = 0.001, d = 0.9) and PF-SMA in the affected hemisphere (*time×group* F_(1,38)_ = 26, *p* < 0.001, d = 0.9) as compared to OGT. Both groups equally improved the remaining deteriorated connectivities found at baseline (all *time×group* interactions *p* > 0.1) and reduced the abnormal hyperconnectivity within SMA, CP, and O ROIs.

**Fig. 9:**
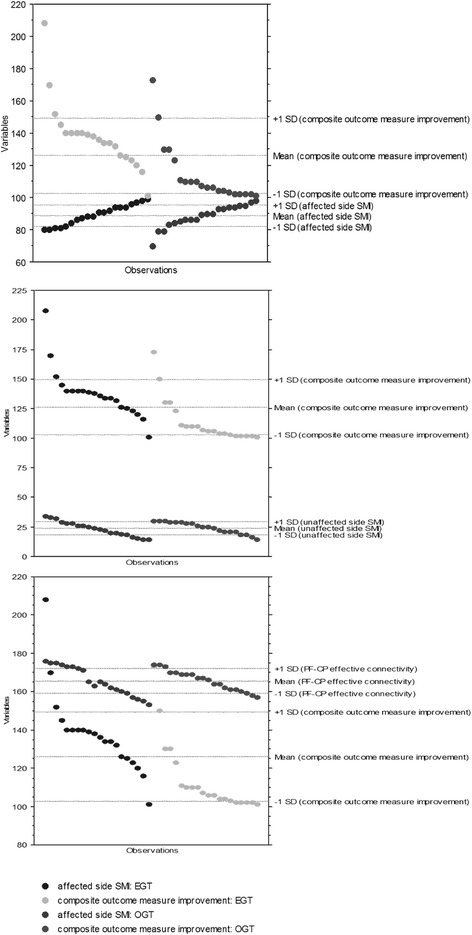
Scatterplot and univariate regression line of electrophysiological outcomes on composite outcome measure (primary) in patients undergoing EGT and OGT

Finally, the multiple regression analysis, with composite clinical outcome measure improvement as a dependent variable, indicated the weakening of SMI in the affected hemisphere (OR 8.5, CI 1.86-38.81, *p* = 0.008, especially in the EGT, Odds 4), the strengthening of SMI in the unaffected hemisphere (OR 36, CI 5.79-223.55, *p* < 0.001, especially in the EGT, Odds 9), and the recovery of PF-CP connectivity (OR 27, CI 4.56-159.66, *p* < 0.001, especially in the EGT, Odds 4) as predictive (independent) variables of clinical improvement. Univariate scattergrams are reported in Fig. 9.

### Discussion

Ekso™ training safely induced a greater improvement in gait velocity, balance, coordination, and performance than conventional gait training. In fact, patients belonging to EGT group got all primary outcomes (namely, the MCID in 10MWT, TUG, and RMI, with medium-to-large effect size), whereas those belonging to OGT got only an MDC. The fact that only Ekso™ significantly improved 10MWT scores is of non-negligible importance, given that walking speed is a cardinal indicator of post-stroke gait performance [53].

These clinical changes were supported by a wide modification of gait measures, including overall gait quality (improved), stance/swing ratio and gait cycle duration (reduced limb asymmetries), and knee flexion (enhanced, as indicated by the amplitude features of RF and BF muscles).

At baseline, patients showed a lower/higher stance/swing ratio in the affected/unaffected limb and an insufficient knee flexion in the paretic side (due to the low activity of BF) paralleled by an abnormal knee flexion in the unaffected side (due to the high activity of non-paretic RF). These changes in sEMG amplitude (which reflects the recruitment and discharge rates of the active motor units and serves as an index of neuromuscular function) represent a compensatory mechanism that maintains the functioning of the paretic limb by increasing the firing rate of the non-paretic muscles, and are aimed to avoid the single support of the paretic side and to increase forward acceleration [54].

Such abnormal patterns were improved more by EGT than OGT. We may argue that Ekso™ positively affected gait by acting onto the terminal stance phase (which has an important role in the knee kinematic control), given that paretic BF and non-paretic RF activations improved significantly in EGT [55]. However, sEMG improvements were limited to proximal muscle whereas both S and TA did not show significant changes. This may depend on the degree of movements provided by the Ekso, which are greater within hip and knee joints.

Therefore, our work confirms previous data on the importance of Ekso™ as an additional treatment to conventional gait training to improve ambulatory functions in chronic post-stroke patients [12]. Indeed, the authors showed that the hip and the total score of Motricity Index, the Functional Ambulation Category, the walking velocity, the distance covered in six minutes, and the number of patients performing the 6MWT and 10MWT improved significantly with the Ekso™ usage. Different from our work, no changes were observed at the knee level, and the improvements in velocity and distance were below the MCID for stroke patients. Nonetheless, there are some differences between ours and the study by Molteni et al. [12] that could account for such discrepancies, including patient sampling, randomization, number, duration and intensity of sessions (higher in our study), and motor task selection.

Other studies using exoskeletons provided contradicting results in patients with chronic stroke [27], showing that EGT is equivalent to traditional therapy for chronic stroke patients, while sub-acute patients may experience added benefit from EGT [56]. In detail, some trials indicated no significant differences in improvement between wearable exoskeleton and overground training groups concerning gait speed, TUG, 6MWT, and 10MWT [26, 56–59]. However, some studies used exoskeleton-stationary rather than exoskeleton-overground walking systems. The strengths of the latter as compared to the former are represented by the high degree of freedom of movement, a lesser extent of the constrainment to the sagittal plane of leg movements, the possibility of multidirectional body movements into the space and of navigating over different surfaces, the different amount of sensory information (including visual, proprioceptive, tactile, and vestibular), and the need that the patient actively interfaces with the exoskeleton to control balance and maintain the trunk [12, 60]. It is, therefore, hypothesizable that exoskeleton-overground walking systems provide the patient with a definitely greater motor control stimulation, multisensory plasticity amount, and required effort to perform the gait training [12].

Other studies indicated that wearable exoskeletons led to a greater improvement then conventional gait training in TUG, 6MWT, and 10MWT [28, 61–64]. Such discrepancies may depend on non-homogeneity in stroke duration, sample clinical-demographic characteristics (e.g., ambulant on ambulant/non-ambulant patients), the device used (AlterG, HAL) and its design (unilateral/bilateral), training period, main walking outcome measures adopted, randomization, and study type (RCT, pre-post study) [27, 56]. Therefore, multicenter randomized controlled trials comparing robotic and conventional over-ground gait training are necessary to confirm that a powered exoskeleton such as the Ekso™ can improve clinical outcome in chronic post stroke patients by finely tuning the gait cycle kinematic.

Our study offers evidence for possible neurophysiological mechanisms harnessed by Ekso™ to induce such clinical improvement. Unilateral brain damage recovery following stroke largely depends on a reshaping of the interhemispheric balance between the CSE and SMI of the affected and unaffected hemispheres mediated by transcallosal inhibition [65–68], and by adaptive changes in FPEC within PF, SMA, and CP regions, including a hyperconnectivity among SMA, CP, and O regions [69], so as to vicariate the loss of neural pathways and to restore the impaired function. Indeed, such regions are all included in the network models supporting motor planning and execution. They include SMA (that plans the coordination of self-paced movements), PF (that oversee motor planning and initiation of motor execution), and CP areas (that are related to the representation and execution of motor programs) [70–72].

Such mechanisms of recovery occur spontaneously and are harnessed by conventional and robotized gait training [14, 15, 73]. However, EGT showed some peculiar neurophysiologic mechanisms. In particular, EGT induced a reshape of CSE of both hemispheres, whereas OGT aftereffects mainly pertained the affected CSE. Moreover, EGT induced a more evident remodulation of SMI between the hemispheres as compared to OGT, given that SMI at TPOST was more balanced following EGT than OGT and that rTMS aftereffects were more evident following EGT than OGT, regardless of the baseline TPOST difference between the groups (that did not affect the magnitude of rTMS aftereffects). These changes were supported by specific variations in FPEC within the unaffected hemisphere, found only in EGT group, with particular regard to the PF-CP and PF-SMA connections, whereas both groups showed an improvement in PF-PF, SMA-CP, CP-CP, and a reduction the of the hyperconnectivity within and toward the posterior regions. Altogether, these effects are in keeping with the specific top-down control of FPEC and the bilateral sensorimotor plasticity exerted by EGT as compared to OGT concerning motor function recovery, with a notable involvement of the unaffected hemisphere [74–76]. The significance of the specific modulations of FPEC and of the rebalance between the SMI concerning the clinical-biomechanical improvement is suggested by the strong correlations among the changes in FPEC, SMI balance, and 10MWT. Moreover, FPEC and SMI balance at baseline predicted 10MWT improvement.

The greater magnitude of the neurophysiological changes induced by EGT, as compared to OGT, may depend on the intrinsic properties of such a wearable device, including a different amount of the sensory inputs (including visual, proprioceptive, tactile, and vestibular). Such a variety of bottom-up information during gait training may significantly affect the top-down modulation of FPEC raised by EGT [77].

There are some limitations to acknowledge. The main limitation consists of the lack of long-term follow-up evaluation. Nevertheless, we have acknowledge that the neurophysiologic mechanisms shaped by neurorobotic rehabilitation may make the re-learned abilities long lasting [13, 14, 17–23], as proven for different RAGT [78]. It is therefore reasonable that powered exoskeletons, including Ekso™, may also offer long-lasting results. This assertion deserves however confirmation in larger follow-up studies, and thus, we have to be cautious in generalizing the results of our study, even though the data are promising.

One may be concerned about the high dose of therapy administered to the patients, which could be a confounding factor when comparing study protocols. However, the dose of the therapy we adopted was the same used in our previous works on RAGT [13, 14].

## Conclusions

Our study suggests that Ekso™ could be useful to promote mobility in persons with stroke owing to mechanisms of brain plasticity and connectivity re-modulation that are specifically entrained by the robotic device, as compared to conventional OGT. Characterizing how top-down connectivity and interhemispheric balance are shaped by neurorobotic therapies could be of remarkable importance to implement patient-tailored rehabilitative training.
